# Withaferin A ameliorates ovarian cancer-induced renal damage through the regulation of expression of inflammatory cytokines

**DOI:** 10.1186/s13048-024-01519-9

**Published:** 2024-10-11

**Authors:** Kusum Kumar, Katherine Bosch, Vasa Vemuri, Nicholas Kratholm, Madhavi Rane, Sham S. Kakar

**Affiliations:** 1 Deparment of Biology, University of Louisville, Louisville, KY USA; 2 Department of Biology and Chemistry, Liberty University, Lynchburg, VA USA; 3 Department of Physiology, School of Medicine, University of Louisville, 500 South Floyd Street, Louisville, KY 40202 USA; 4 Department of Medicine, Division Nephrology, University of Louisville, Louisville, KY USA; 5 Department of Medicine, Brown Cancer Center, University of Louisville, Louisville, KY USA

**Keywords:** Kidney damage, Renal dysfunction, Creatinine, Inflammatory cytokines, Ovarian cancer and cachexia

## Abstract

**Background:**

Cachexia a multifactorial syndrome is a common sequala in patients with cancer. It varies from 42 to 80% depending upon the oncological stage and is directly responsible for 30% of deaths in these patients. Previous research from our laboratory demonstrated that peritoneal ovarian cancer generated in NSG mice resulted in skeletal and cardiac muscle atrophy - leading to loss of skeletal muscle mass and strength, and cardiac dysfunction (cachexia). Treatment of mice bearing i.p. tumors with withaferin A (WFA) showed reversal of skeletal muscle and cardiac cachexia. The present study is focused on determining effects of peritoneal ovarian tumors on kidney damage and effects of WFA treatment on ameliorating kidney damage.

**Methods:**

We generated intraperitoneal ovarian cancer by injecting female NSG mice with ovarian cancer cell line (A2780). After one week of injecting cancer cells, mice were treated with WFA (4 mg/kg) every third day, for three weeks. After 4 weeks of injection of cancer cells, the mice were sacrificed and various tissues including kidney and blood were collected, snap-frozen in liquid nitrogen, and stored at -80^0^C. The presence of kidney biomarker creatinine, was measured in the plasma by an ELISA. The mRNA was isolated from mouse kidneys and was used to examine the expression levels of signaling proteins, inflammatory cytokines, and genes responsible for inducing cachexia (IL-1β, IL-6, TNF-α, TGF-β, GDF-15, and MYD88).

**Results:**

Our results showed a significant increase in levels of expression of inflammatory cytokine IL-1 β (*p* < 0.01), IL-6 (*p* < 0.001), TNF-α (*p* < 0.001), and other related genes including TRAF6 (*p* < 0.01), MYD88 (*p* < 0.01), and GDF-15 (*p* = 0.005) in tumor-bearing mice compared to controls. Treatment of mice bearing tumors with WFA attenuated the increase in expression of each gene. In addition, our results showed a significant increase in creatinine levels in circulation in tumor-bearing mice compared to control mice. Treatment of tumor-bearing mice with WFA resulted in a significant decrease in plasma creatinine levels compared to tumor-bearing mice.

**Conclusions:**

Our results conclude that ovarian tumors in NSG mice caused kidney damage and renal dysfunction, which was effectively ameliorated by WFA treatment, suggesting a protective effect of WFA on kidney injury induced by ovarian cancer.

## Introduction

Cachexia is a complex syndrome frequently observed in cancer patients, with its occurrence ranging from 42 to 80% depending on the oncological status. It accounts for approximately 30% of deaths in these patients [1, 2]. Cachexia can lead to poor appetite, weight loss, and a decline in muscle strength [3, 4]. In recent years, investigators have shown that cancer cachexia implicates not only the loss of skeletal muscle, but also causes significant pathologic remodeling of several organs including heart, liver and adipose tissues, and pancreas [5, 6]. Several mechanisms and signaling pathways leading to development of cachexia have been reported. However, a definitive orchestrating mechanism remains elusive [6]. Further, no FDA approved therapeutic regimen for cancer-induced cachexia is currently available.

In our previous studies, we explored the role of Withaferin A in ameliorating cancer-induced muscle and cardiac cachexia [7, 8]. Withaferin A is a steroidal lactone that is a purified extract from the *Withania somnifera* plant, commonly referred to as winter cherry or Ashwagandha [9]. WFA is known for its anticancer, anti-inflammatory and cardiac protective properties [10–12]. In our previous investigations, we revealed that injection of ovarian cancer cells A2780 into peritoneal cavity developed tumors and encapsulated the clinical features of muscle cachexia including loss of skeletal mass and strength. Treatment of mice bearing ovarian tumors with WFA (2 mg/kg or 4 mg/kg) reverted the loss of muscle mass and strength, and completely mitigated the slow-to-fast myofiber type conversion typically observed in the context of cancer cachexia [12]. Our study also showed that treatment of mice with WFA led to a reduction of NF-κB-related pro-inflammatory cytokines and NLRP3 inflammasome signaling [12]. Cardiac cachexia is a complication of inflammatory diseases leading to chronic heart failure (CHF) and is associated with muscle wasting and a poor clinical prognosis. Recent research indicates that the prevalence of cardiac cachexia varies between 8 and 42% depending on the cancer state [13]. The release of inflammatory cytokines by tumors initiates the pathogenesis of heart failure, and the chronic inflammation further contributes to the development of heart failure [14–16]. In our studies, we showed that generation of tumors in NSG mice injected with ovarian cancer cells (A2780) not only induced muscle cachexia but also cardiac cachexia. This was confirmed by a decline in cardiac performance, as evidenced by a significant reduction in parameters such as heart weight, heartrate, fractional shortening, ejection fraction, cardiac output, and left ventricular mass [7]. The administration of WFA in tumor-bearing mice yielded substantive outcomes, notably marked by a restoration of the myofibrillar cross-sectional area along with a complete amelioration of cardiac dysfunction [7]. Based on our studies, we hypothesize that cancer induces kidney damage, and such damage can be reverted by treatment with WFA. The present study aims to elucidate the specific pathways involved in cancer-induced kidney damage (cachexia) especially inflammatory cytokines in a mouse model, and to test the efficacy of WFA in reversal of kidney damage.

## Materials and methods

### Generation of ovarian tumor in mice followed by treatment with withaferin A

Female NOD.Cg-Prkdc^scid^ Il2rg^TM1Wjl^/SzJ (NSG, Jackson Lab Strain # 005557) immunodeficient mice (5 to 6 weeks old) were purchased from Jackson Laboratory. After one week of assimilation, mice were randomized into two groups (a tumor-bearing and tumor-free, 20 mice/group). The tumor-bearing mice were injected (intraperitoneal) with ovarian cancer cells (A2780) (8.0 × 10^5^ cells suspended in 100 µl of PBS), while the tumor-free mice received i.p. injection of 100 µl sterile saline. After one week of implantation of cells, both tumor-free and tumor-bearing groups were stratified into a group that received vehicle injections (10% dimethyl sulfoxide, 90% glycerol trioctanoate) or WFA (4 mg/kg) via i.p. injection (10 mice/group) on every third day for three weeks. After four weeks of implantation of tumor cells, mice were scarified, kidney, other tissues and blood were collected. Plasma was prepared from each blood sample. Tissues and plasma samples were stored at -80^0^C for biochemical analysis.

### RNA purification

Kidney was sliced and a small piece was snap frozen for total RNA isolation. Total RNA was isolated from kidneys using Qiagen RNeasy Plus Mini Fibrosis Tissue Kit (Cat # 74104). The purity and RNA quality was validated by performing agarose gel electrophoresis. Total RNA from each sample was quantitated using nano-drop spectrophotometer as described previously [17]. One µg of total RNA from each sample was used to synthesize first strand cDNA using the Bio Rad iScript cDNA synthesis kit (Cat #1708891).

### Quantitative PCR (qPCR)

The cDNA from each sample was used to evaluate the expression levels of various genes using quantitative polymerase chain reaction (qPCR), using the SYBR green, and specific primers for each gene (Table 1) using a Bio-Rad CFX Connect Real-Time PCR as described previously [17]. Beta actin primers were used to amplify β-actin gene as a control to normalize levels of expression of each gene.

### Statistical analysis

Primary statistical analysis was performed to determine fold-change for each gene compared to the gene expression of our baseline gene, β-Actin. Data analysis was performed using the Graph Pad Prism software’s two-way ANNOVA and post-hoc Tukey’s test, with p values of *p* < 0.05 were considered to be significant.

### Ethics statement

All procedures involved in usage of mice was carried out in strict accordance with the standards of the National Institute of Health guideline for the care and use of laboratory mice. The Institutional Animal Care and Use Committee (IACUC, protocol # 15405) and Institutional Biosafety Committee (IBC, protocol # 18–208) of the University of Louisville approved all experimental protocols in mice in advance. No human data or tissue was used in the present study.

**Table 1:** Primer sequences used in qPCR

Gene	Forward Sequence	Reverse Sequence
β-Actin	CAGGCATTGCTGCAGGATG	TGCTGATCCACATCTGCTGG
GDF-15	GAGCTACGGGGTCGCTTC	GGGACCCCAATCTCACCT
IL-1 β	ACAGATGAAGTGCTCCTTCCA	GTCGGAGATTCGTAGCTGGAT
IL-6	ACACAGACAGCCACTCACCT	TTCTGCCAGTGCCTCTTTGC
MYD-88	ACCTGTGTCTGGTCCATTGCCA	GCTGAGTGCAAACTTGGTCTGG
TNF-α	CCCAGGGACCTCTCTCTAATC	ATGGGCTACAGGCTTGTCACT
TRAF-6	TCGCCTAGTAAGACAGGACCATCA	TGGTTCCATTTCGGCAACCT
TGF-β	TGATACGCCTGAGTGGCTGTCT	CACAAGAGCAGTGAGCGCTGAA

## Results

### WFA significantly restores blood creatinine to homeostatic levels

The glomerular filtration rate (GFR) is one of the most effective indexes to evaluate renal functional decline and chronic kidney disease [18]. The serum/plasma creatinine levels are commonly used to evaluate GFR. This biomarker is effective due to its low variations and its ability to be freely filtered from the glomerulus. The most common method for creatinine measurements is the Jaffe-based creatinine-picrate formed in an alkaline medium. Chronic Kidney Disease (CKD) can lead to the inability of kidneys to filter blood appropriately.

**Fig. 1:**
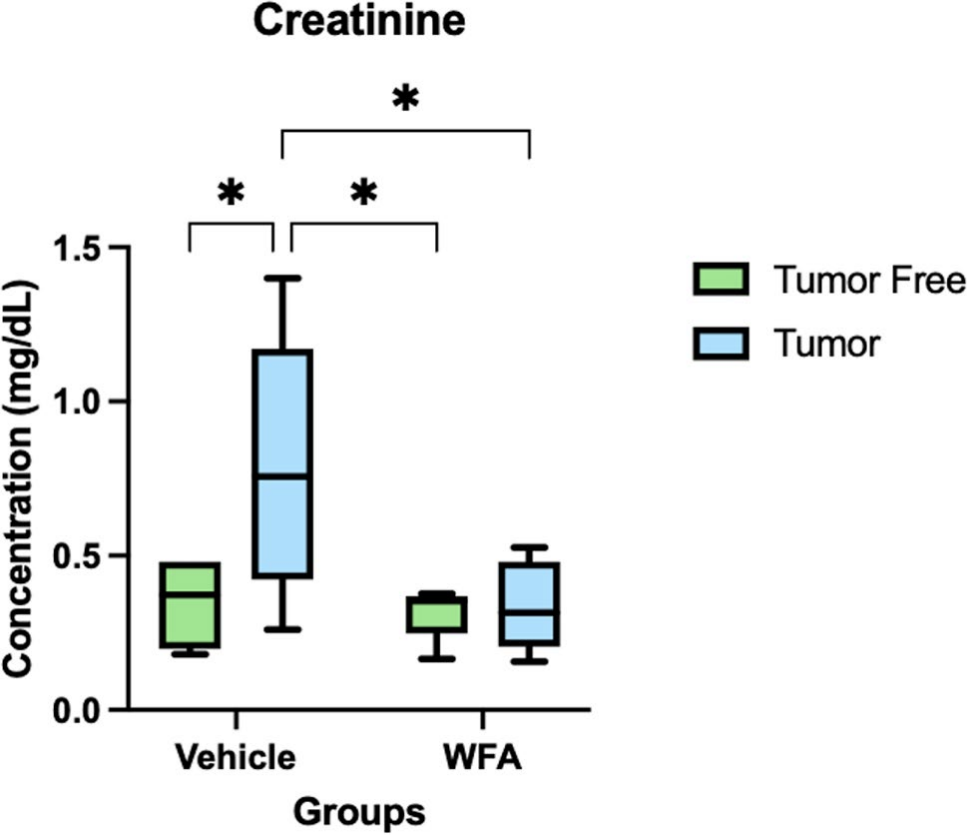
Measurement of plasma creatinine levels in tumor bearing and tumor free groups. *N* = 6 for all the groups. *p < 0.05 value is significantly different from tumor-free vehicle (control) group and the tumor-vehicle group by two-way ANOVA followed by Tukey’s multiple comparison test post hoc analysis

In our studies, we observed that control group of mice (tumor-free and injected with vehicle) showed very low levels of creatinine in blood plasma. However, significantly increased plasma creatinine levels were detected in the tumor-bearing vehicle group, suggesting a defect in GFR and indicative of renal dysfunction. Treatment of tumor-free mice with WFA showed no difference in creatinine levels compared to control mice injected with vehicle (Fig. 1). However, increase in creatinine levels in tumor bearing mice were significantly reduced by treatment with WFA. A significant increase in creatinine levels in tumor bearing mice indicates that ovarian tumors generated in mice induced kidneys damage resulting in decrease of GFR and such damage was reversed by treatment with WFA.

### WFA restores the levels of inflammatory cytokines

Tumors cause chronic inflammation throughout the body, inhibiting anti-tumor immune response mounted by the body, allowing the cancerous cells to proliferate. This inflammation is controlled by inflammatory cytokines. One of the major cytokines involved in this inflammation is IL-1β, which promotes tumor growth and invasiveness through the stimulation of IL-6, TGFβ, and TNFα. IL-1β is fully activated in two steps. First is a priming step, where IL-1β is produced as an inactive 31 kDa precursor, namely pro-IL-1β, in response to inflammasome activation [19]. Second, the pro-IL-1β is cleaved and activated by active Caspase-1 [20, 21]. Caspase-1 independent activation of IL-1β is also documented [22]. Once active, IL-1 β binds to the IL-1R, which binds to MyD88 creates a complex with IRAK1, IRAK4, TRAF-6, and TAK1 [23]. Toll-like receptor 4 (TLR4) operates through a similar pathway to IL-1R.

**Fig. 2:**
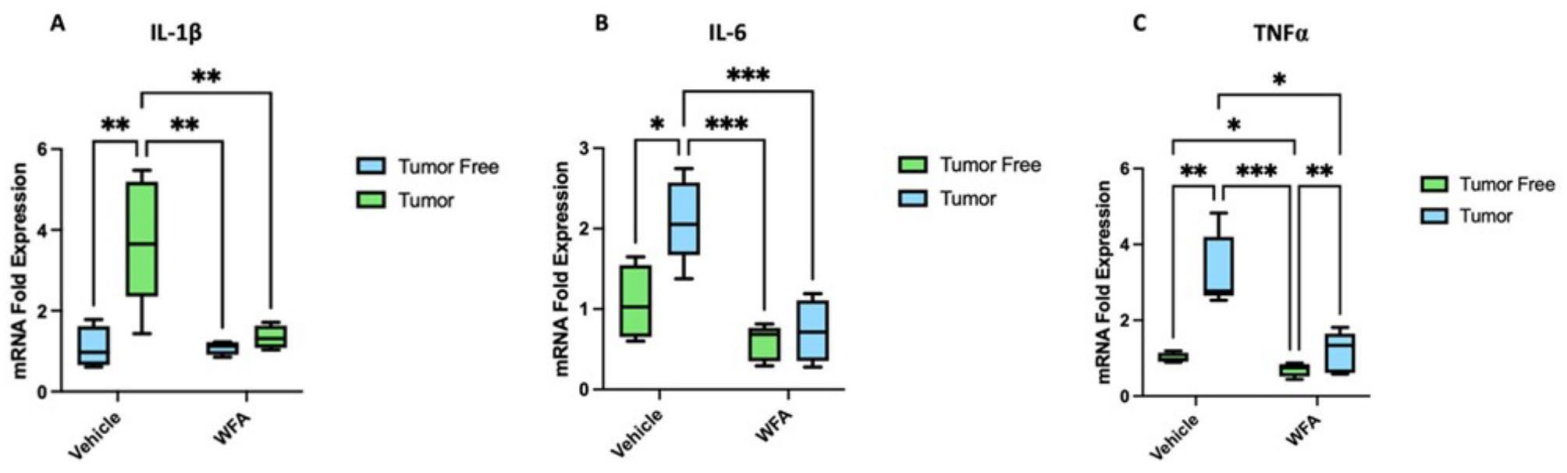
Expression of IL-1ß, IL-6, and TNFα cytokines mRNA in kidney tissues. Levels of expression of IL-1β (2A), IL-6 (2B), and TNFα (2C)cytokines showed a significant increase in tumor bearing mice and expression levels were significantly reduced when treated with WFA. *p < 0.05, **p < 0.01, ***p < 0.001

We examined the expression of IL-1β cytokine in control and tumor-bearing groups. The fold change of IL-1β was measured between the groups (Fig. 2A). Tumor bearing mice showed a significant increase in expression of IL-1 β levels as compared to the vehicle-treated control tumor-free mice (*p* < 0.01). Treatment of tumor bearing mice with WFA showed a significant reduction in IL-1β expression levels (*p <* 0.001). WFA by itself did not modulate IL-1β expression in control tumor-free mice, as no significant difference in IL-1β expression was observed when these mice were treated with vehicle or WFA.

The other important inflammatory cytokines involved in the kidney damage is IL-6. Production of IL-6 often occurs at the site of inflammation and can be induced by IL-1β, angiotensin, LPS, and TNF-α through the TRAF-6 signaling pathway. IL-6 increases the production of T and B cells, which play important role in transitioning tissues from acute inflammation to chronic inflammation. Acute inflammation typically occurs through the production of inflammatory cytokines. These cytokines stimulate acute-phase proteins produced by hepatocytes. Stimulation of IL-6 acts to suppress some of these pro-inflammatory cytokines to control the amount of inflammation. However, under certain conditions, IL-6 stimulation can lead to B-cell differentiation, T-cell activation, and the recruitment of monocytes to the site of inflammation. These changes mark the switch from acute inflammation to chronic inflammation. Because of important role of IL-6 in regulation of inflammation leading to kidney dysfunction, we measured the IL-6 mRNA levels in kidney tissues using the specific primers in qPCR. As shown in Fig. 2B, levels of IL-6 mRNA were found to be significantly (*P* < 0.05) upregulated in tumor bearing mice compared to control tumor-free mice. Treatment of tumor bearing mice with WFA resulted in a significant decrease (*p* < 0.001) in IL-6 levels matching levels observed in tumor-free mice. WFA did not alter IL-6 mRNA expression in tumor-free mice, as no significant difference in IL-6 mRNA expression was detected under WFA or vehicle treatment in these mice. Our results suggest that increase in IL-6 expression levels by ovarian cancer may lead to kidney inflammation and its reversal by WFA.

Another inflammatory cytokine, TNFα, has been reported to be expressed throughout the kidney including podocytes, mesangial cells, and epithelial cells. The production of TNFα can be induced by lipopolysaccharides, angiotensin II, hypertension, and renal failure. In addition, the IL-1β/TLR4-MyD88-TRAF-6 pathway can stimulate TNFα. TNFα promotes cytokine release, inducing inflammation, leading to kidney damage [24]. TNFα binds to TNF receptors (TNFR), which forms a complex with TRADD, TRAF2, and RIP. This complex activates JNK, promoting the production of ROS and induction of apoptosis. However, in the presence of TRAF6, TNFR can activate NF-kB. In this proposed mechanism, TRAF6 activates PI3K, Akt, and GSK3β, which eventually leads to NF-kB activation and inhibition of TNF-α induced apoptosis by inhibiting ROS and JNK activation [25]. As shown in Fig. 2C, TNFα, was found to be highly expressed in tumor bearing mice. The expression levels of TNFα were found to be significantly (*p* < 0.01) higher in tumor bearing mice compared to tumor-free mice. Such increase in TNFα in tumor bearing mice were significantly decreased on treatment with WFA (*p* < 0.001). A significant (*P* < 0.05) difference was also observed in tumor-free mice when treated with WFA compared in tumor-free mice treated with vehicle suggesting beneficial effect of WFA on down regulation TNFα in kidney.

**Fig. 3:**
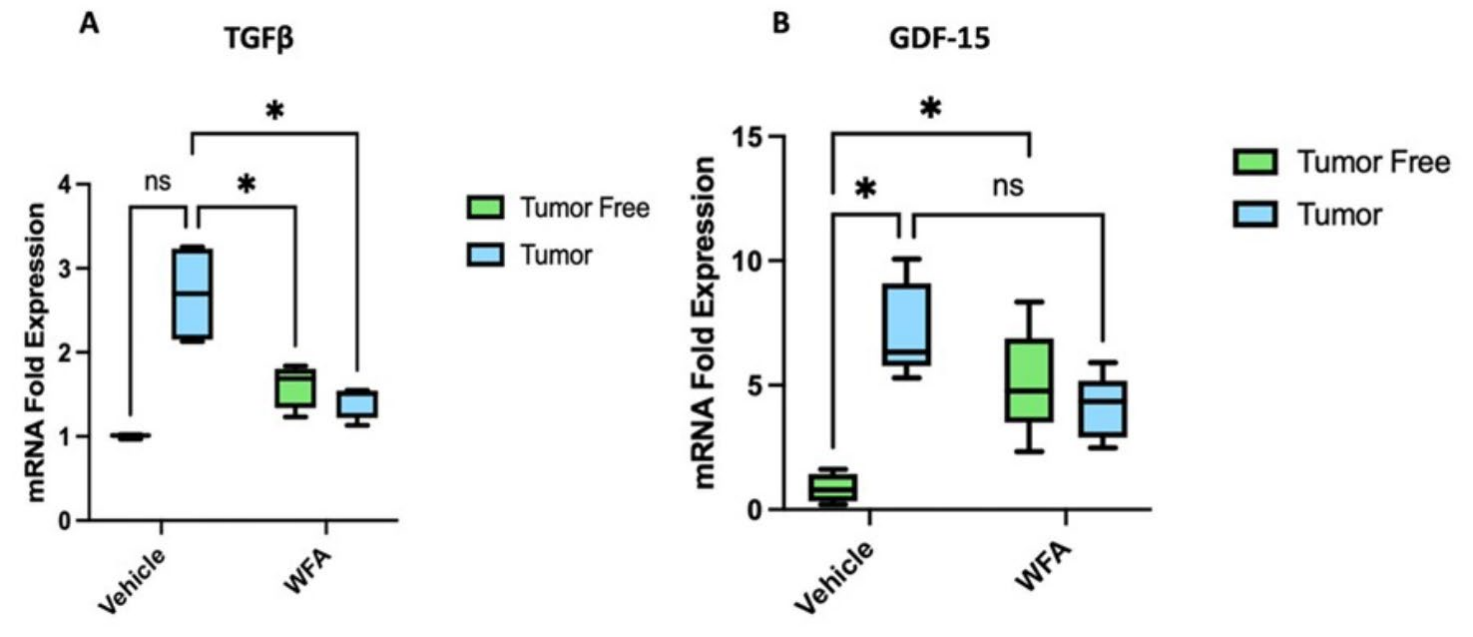
Expression levels of TGFβ and GDF-15 in tumor bearing and tumor free mice. **A**: a nearly significant increase in TGF-β mRNA expression levels in tumor bearing mice, and a significantly decrease in expression levels on treatment with WFA. **B**: GDF-15 expression levels significantly increased in tumor bearing mice compared to tumor free mice. *p* < 0.05, **p < 0.01, ***p < 0.001

TGFβ is a protein involved in the transcription of inflammatory cytokines and promotes fibronectin and collagens. The increased transcription of fibronectins and collagens is directly linked to fibrosis. TGFβ works through multiple pathways to achieve the increased transcription of fibronectin and collagen. One such TGFβ pathway studied involves TRAF-6. Binding of TGFβ to its receptor complex including TGFβRI and TGFβRII, activates TRAF-6. TRAF-6 along with TAK1 can activate NF-kB, JNK, and p38 in a similar mechanism to the TNFα-TRAF6 signaling cascade [26]. However, TGF-β can act independent of TRAF6 through a Smad signaling pathway. In this pathway, once the TGFβRI and TGFβRII complex is formed, Smad2/3 is phosphorylated and forms a complex with Smad4. This Smad2/3/4 complex can act to promote the transcription of collagens [27] leading to fibrosis. Although TGF-β expression in tumor bearing mice trended higher as compared to tumor-free mice, it failed to reach significance (*p* = 0.0617). Treatment of tumor bearing mice with WFA resulted in a significant reduction in TGF-β expression levels compared to tumor bearing mice treated with vehicle (Fig. 3A). No significant difference was observed between tumor-free mice treated with vehicle and WFA respectively. These results suggest that tumor induces fibrosis in kidney leading to defect in renal system and WFA attenuates such deleterious effects. Future studies will elucidate tumor-induced kidney fibrotic pathways in these mice.

Growth differentiation factor 15 (GDF-15) also known as MIC-1 is a multifunctional cytokine that belongs to the superfamily of transforming growth factor-beta (TGFβ). GDF-15 is involved in immune tolerance and is elevated in several acute and chronic stress conditions, often correlating with disease severity and patient prognosis in cancer and metabolic and cardiovascular disorders [28]. Its levels have been shown to be upregulated in patients with cancer cachexia, as well as in animal models of pancreatic, colon, head and neck, breast and prostate cancers [29–32]. Elevated circulating levels of GDF-15 may lead to anorexia, weight loss and decreased in survival in patients with cancer cachexia [31]. Because of importance of GDF-15 in cancer induced cachexia, we measured its expression in tumor bearing and tumor free mice. In our studies, we showed a significant (*p* < 0.05) increase in expression of GDF-15 levels in tumor bearing mice compared to tumor-free mice. Treatment of tumor bearing mice with WFA showed a decrease in GDF-15 levels. However, statistically significant difference was not seen (*p* < 0.076) (Fig. 3B). These results strongly suggest that increase of GDF-15 expression in tumor mice may play a role in kidney cachexia.

### Expression of signaling mediators

**Fig. 4:**
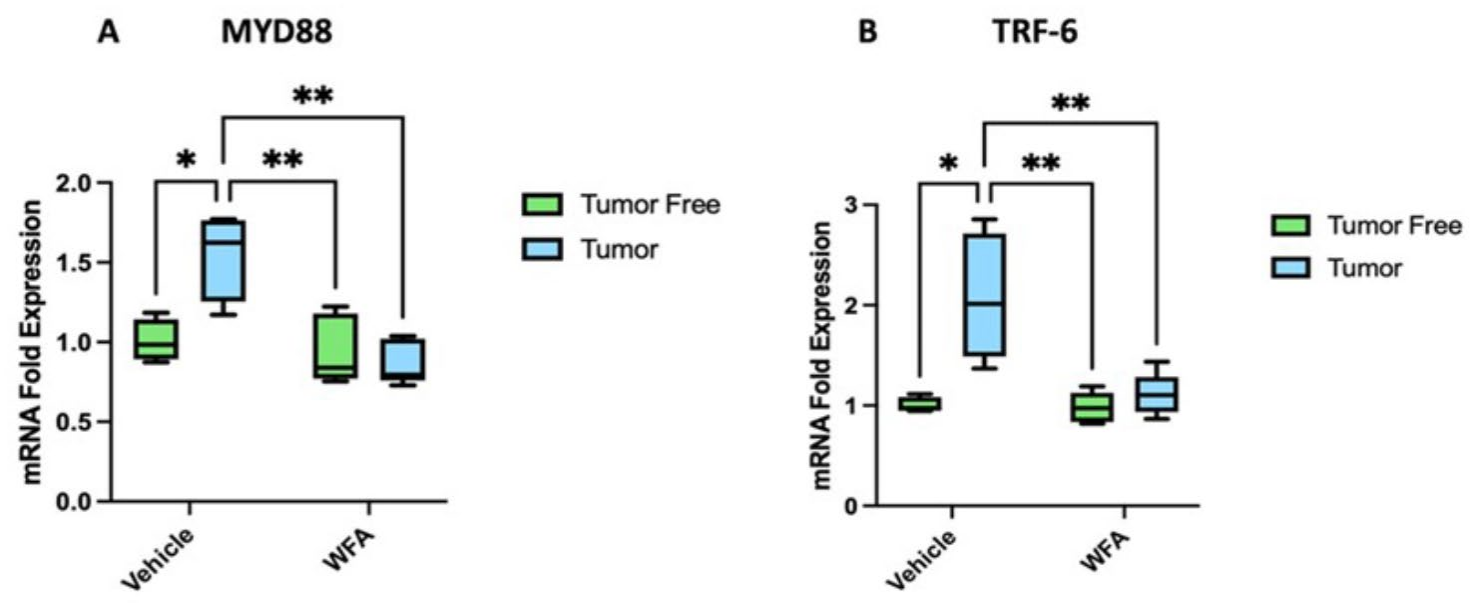
MyD88 (**A**) and TRAF-6 (**B**), key mediators of the inflammatory cytokine signaling pathway, both show significant increases in expression levels in tumor bearing mice compared to tumor free mice. Increase in MyD88 and TRAF-6 expression levels in tumor bearing mice decreased to normal levels on treatment of mice with WFA. *p < 0.05, **p < 0.01, ***p < 0.001

MyD88 and TRAF-6 are key signaling mediators for TLR and IL-1β signaling pathways. The fold change of each of these cytokines was measured between groups (Fig. 4). Analysis of MyD88 expression showed a statistically significant increase of MyD88 expression levels in tumor-bearing group compared to control group (*p* < 0.05). Tumor-bearing group when treated with WFA showed a statistically significant reduction in MyD88 expression levels compared to the tumor-bearing group (*p* < 0.01) (Fig. 4A). No significance difference in the levels of expression between control groups (tumor-free mice treated with vehicle and control tumor free treated with WFA) was observed. Similarly, TRAF-6, gene expression showed a statistically significant increase in the tumor-bearing group compared to control (*p* < 0.01) (Fig. 4B). Treatment of tumor bearing mice with WFA showed a significant decrease when compared to the tumor-bearing group (*p* < 0.01). No significant difference was seen in control mice treated with either vehicle or WFA (Fig. 4).

## Discussion

Cancer is known to induce damage to various organs leading to organ fibrosis and dysfunctions also known as cachexia. To the best of our knowledge, there is no FDA approved drug available for the treatment of cachexia. In our previous studies [7, 8] we demonstrated the attenuation of cancer-induced muscle and cardiac cachexia by withaferin A (WFA). To the best of our knowledge, there is no report in reversal of cancer-induced renal damage (cachexia) by WFA. Creatinine is clinically used as a surrogate marker of kidney function. Increase in levels of creatine in patient’s blood or urine is an indication of renal dysfunction [33]. The current study utilized the Jaffe-based creatinine-picrate formation in an alkaline medium as a method to measure creatinine levels in plasma. The estimated glomerular filtration rate estimates how effectively the kidney filters certain agents from the body. By comparing the experimental results of the eGFR to a generally accepted baseline, one can discover a disturbance in the kidney’s filtering ability, which can be a cause of diseases such as chronic kidney disease. In the present study, we observed a significant increase plasma creatinine levels in tumor bearing mice indicating a significant renal damage that caused the inability of the kidneys to function properly. On treatment of tumor bearing mice with WFA, the plasma creatine levels returned to the homeostatic levels, suggesting that the WFA treatment is an effective treatment in restoring the filtering ability of the kidneys and by reversing kidney damage induced by cancer.

Renal fibrosis can lead to kidney failure.  Fibrosis and kidney damage can be caused by the activation of proinflammatory cytokines and their signaling pathways. Our results indicate that mice with tumors displayed increased in gene expression of several of a number of these proinflammatory cytokines. Prolonged overexpression of tumor-derived inflammatory cytokines initiates muscle and cardiac cachexia, as shown by previous studies [7, 14–16, 34]. This suggests that ovarian cancer has a direct effect on the kidney’s function, as the tumor led to increase in expression levels of IL-1β, TNFα, IL6, and GDF-15. Treatment of mice bearing tumor with WFA, however, normalized the levels of these inflammatory cytokines, suggesting protective effects of WFA on kidney damage induced by cancer. These results are consistent with our previous studies on induction of muscle cachexia [12] and cardiac cachexia [7] and their reversal/protection by WFA treatment. The combination of these studies show that the results are consistent amongst previous experiments in our lab.

Measurement of expression of GDF-15 gene showed significant increase in expression levels in tumor bearing mice. However, the treatment with WFA did not significantly decrease GDF-15 to baseline.  The gene expression levels of TGFβ increased without significant in tumor bearing mice. However, treatment with WFA resulted a significant decrease in TGFβ levels. When GDF-15 levels are neutralized in a cancerous mouse model, it is shown to restore muscle mass and muscle function lost during cachexia [35]. Expression of both GDF-15 and TGF-β have been reported to significantly increased in chronic kidney disease [36] and in mice infused with angiotensin II.

In our studies [7] we showed a significant increase in Ang II in circulation in tumor bearing mice, and its normalization on treatment with WFA. These results suggest that damage caused by tumor in part could be attributed to increase in levels of Ang II in circulation and its reversal by WFA.

In addition to investigate the changes in expression levels of inflammatory cytokines in kidney, we also analyzed the expression levels of two key signaling mediators, MyD88 and TRAF6. MYD88 is involved in TLR and IL-1β [37] intracellular signaling, and TRAF6 is involved in TLR, IL-1, TGF-β, and TNF-α signaling [38]. Our results showed that the gene expression of both MyD88 and TRAF6 increased significantly in the tumor-bearing groups when compared to the control groups. However, when treated with WFA, the gene expression levels of both mediators returned to normal levels. These results suggests that ovarian cancer-induced renal damage is propagated by the overexpression of inflammatory cytokines, and the overexpression of signaling mediators genes.

## Conclusions

**Fig. 5:**
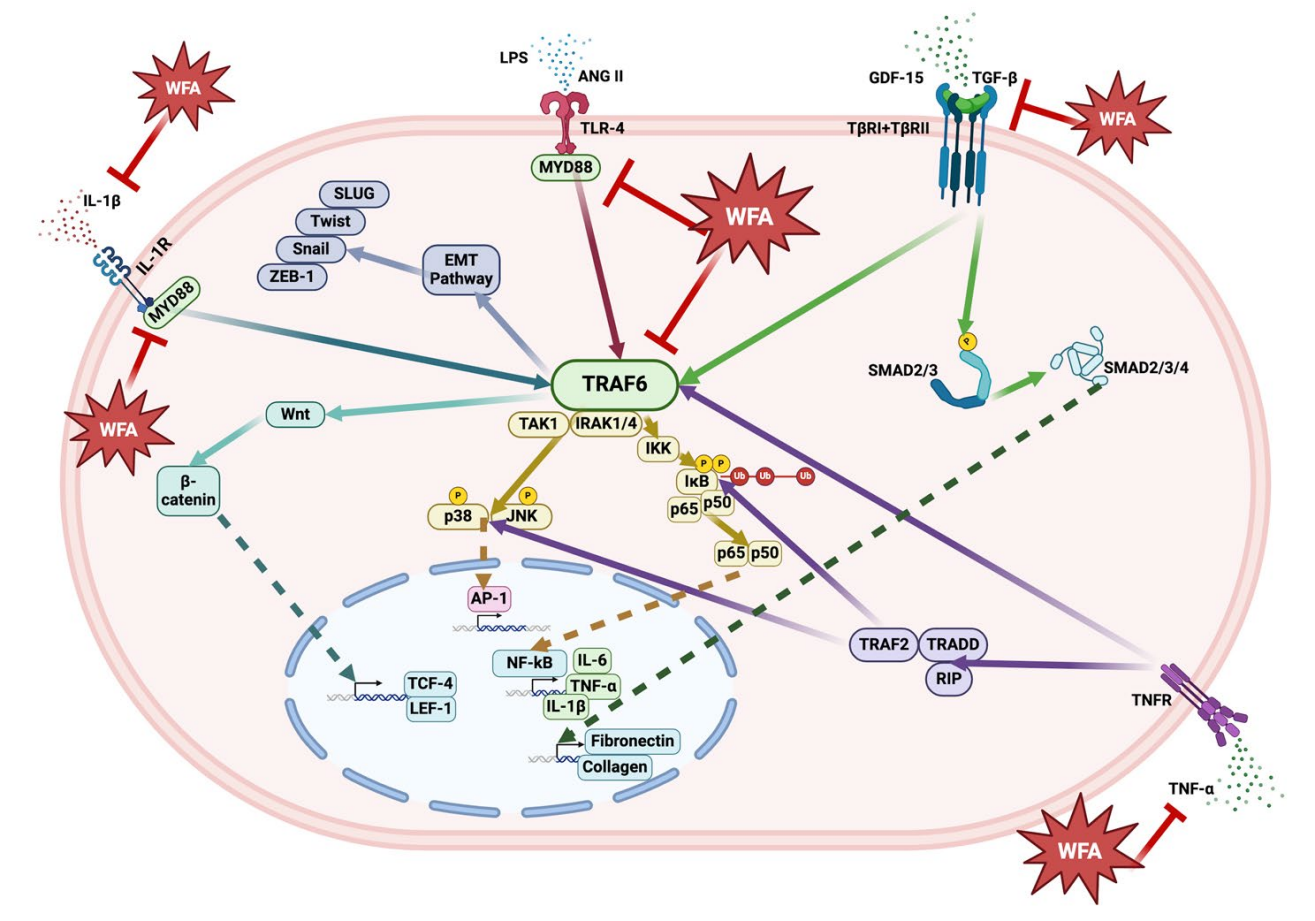
The proposed signaling pathway illustrating effects of cancer and inflammation in the kidney. TRAF6 and MyD88 activation through IL-1β, TGFβ, TNFα, angiotensin II, LPS, and GDF-15 leads to the activation of transcription of renal damage-causing genes. WFA inhibits the expression of IL-1β, TNF-α, TGF-β, MyD88, and TRAF6. Figure created with Biorender

To the best our knowledge, our studies for the first time showed that the generation of ovarian cancer in NSG mice caused a significant increase in plasma creatinine levels indicating induction of renal dysfunction (cachexia). Kidney damage induced by ovarian cancer was reversed on treatment of tumor bearing mice with WFA. Analysis of various inflammatory cytokines and signaling genes IL-1β, TNFα, IL-6, and GDF-15, MyD88, and TRAF-6 known to induce kidney dysfunction and fibrosis showed a significant increase in the expression levels in kidney in mice bearing tumors. Treatment of mice bearing tumors with WFA normalized the levels of expression of these genes to control levels. These results suggest that WFA reverses the kidney damage leading to renal dysfunction caused by ovarian cancer and regulating the overexpression of proinflammatory cytokines (Fig. 5).

## Data Availability

Datasets used and/or analyzed during the current study are available from the corresponding author on reasonable request.
